# Phylogenetic relationships of three rockfish: *Sebastesmelanops*, *Sebastesciliatus* and *Sebastesvariabilis* (Scorpaeniformes, Scorpaenidae) based on complete mitochondrial genome sequences

**DOI:** 10.3897/BDJ.11.e98167

**Published:** 2023-02-28

**Authors:** Peter C. Searle, Andrea L. Kokkonen, Jillian R. Campbell, Dennis K. Shiozawa, Mark C. Belk, R. P. Evans

**Affiliations:** 1 Department of Biology, Brigham Young University, Provo, UT, United States of America Department of Biology, Brigham Young University Provo, UT United States of America; 2 Department of Microbiology and Molecular Biology, Brigham Young University, Provo, UT, United States of America Department of Microbiology and Molecular Biology, Brigham Young University Provo, UT United States of America; 3 Monte L. Bean Life Science Museum, Brigham Young University, Provo, UT, United States of America Monte L. Bean Life Science Museum, Brigham Young University Provo, UT United States of America

**Keywords:** *Sebastes*, *s*peciation, phylogenetics, rockfish, mitogenome

## Abstract

We characterise the complete mitochondrial genomes (mitogenomes) of Black rockfish (*Sebastesmelanops* Girard, 1856; n = 1), Dark rockfish (*Sebastesciliatus* Tilesius, 1813; n = 2) and Dusky rockfish (*Sebastesvariabilis* Pallas, 1814; n = 2). The lengths of the mitogenomes are 16,405 bp for *S.melanops*, 16,400 bp for both *S.ciliatus* and 16,400 and 16,401 bp for *S.variabilis*. We examine these species’ phylogenetic relationships using 35 previously published rockfish mitogenomes, representing 27 species. We find that *S.melanops* is sister to a clade consisting of *S.rubrivinctus*, *S.nigrocinctus*, *S.umbrosus* and *S.oculatus*, whereas *S.ciliatus* and *S.variabilis* are sister to a clade consisting of *S.norvegicus*, *S.viviparus*, *S.mentella* and *S.fasciatus*. We were unable to separate *S.ciliatus* and *S.variabilis* using their complete mitogenomes.

## Introduction

**Figure 1.**
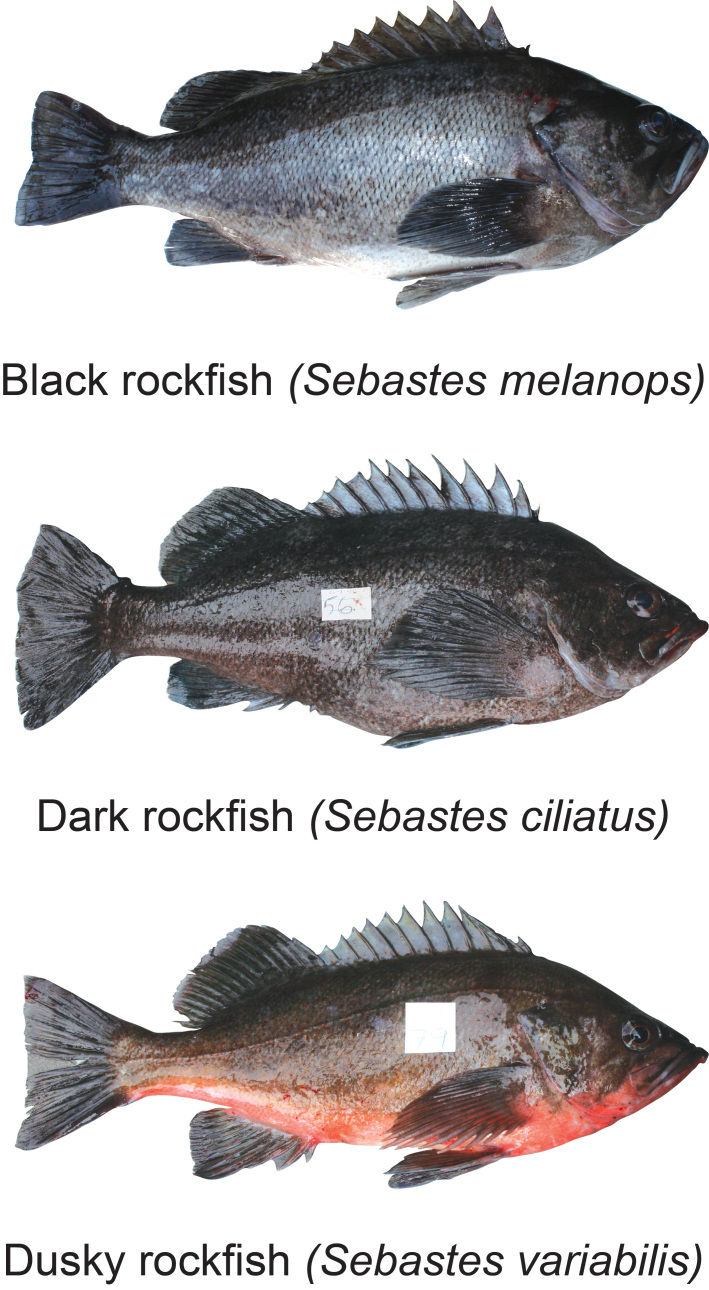
Photographs of Black rockfish (*Sebastesmelanops*), Dark rockfish (*Sebastesciliatus*) and Dusky rockfish (*Sebastesvariabilis*). Photos taken by Mark C. Belk.

Black rockfish (*Sebastesmelanops* Girard, 1856), Dark rockfish (*Sebastesciliatus* Tilesius, 1813) and Dusky rockfish (*Sebastesvariabilis* Pallas, 1814) are members of *Sebastes* (Cuvier, 1829), a diverse genus of marine fishes comprising more than 110 species (Fig. 1). These commercially important rockfishes are found in the North Pacific Ocean, with sympatric geographic ranges. *Sebastesmelanops* schools over high relief rocky outcrops from 0-366 m, *S.ciliatus* schools over high relief on rocky reefs and in kelp forests from 5-160 m and *S.variabilis* schools over high-relief sea floors from 6-675 m (Butler et al. 2012).

Although *S.ciliatus* and *S.variabilis* were described separately in the early 1800s, they have long been considered a single variable species under the name *S.ciliatus* (Jordan and Gilbert 1881, Eigenmann and Beeson 1894). However, the presence of two colour morphs within *S.ciliatus*, with associated ecological differences, led to speculation that *S.ciliatus* consisted of a dark, shallow-water morph (*S.ciliatus*) and a light, deep-water morph (*S.variabilis*; Eschmeyer and Herald (1983), Kessler (1985)). Orr and Blackburn (2004) officially resurrected *S.variabilis* from *S.ciliatus* using morphological and meristic data, but molecular analyses have produced conflicting results. Genetic differences were identified in *S.ciliatus* using allozymes (Tsuyuki et al. 1965, Seeb 1986) and microsatellites *(Orr and Blackburn 2004*); however, it is unclear if these differences resulted from species-level separation or population-level differences resulting from geographic separation of the samples. Tsuyuki et al. (1965) did not provide specific location data for their nine samples of *S.ciliatus* and the samples analysed by Seeb (1986) did not come from sympatric populations. Conversely, mitochondrial DNA, specifically NADH dehydrogenase subunits, was not significantly different (Orr and Blackburn 2004). We report the complete mitogenomes of *S.melanops*, *S.ciliatus*, and *S.variabilis* to provide new insight into the taxonomic relationships amongst these species. We aimed to determine if the lack of resolution in mitochondrial DNA was limited by the small portion of mitochondrial DNA examined in previous studies.

## Materials and Methods

Using hook-and-line sampling, we collected three rockfish specimens (*Sebastesmelanops*, *S.ciliatus* and *S.variabilis*) from Frederick Sound, near Admiralty Island (57.307504, -134.133069) in 2018 and two rockfish specimens (*S.ciliatus* and *S.variabilis*) near Excursion Inlet, Alaska (58.3159, -135.4592) in 2019 (IACUC-approved protocol #15-0602). Upon capture, we euthanised specimens with tricaine methanesulfonate (MS-222, MilliporeSigma, St. Louis, MO, USA), excised liver samples and placed the samples in RNAlater (MilliporeSigma, St. Louis, MO, USA). Samples were flash-frozen at -20°C, transported to Brigham Young University and stored at -80°C. Samples were catalogued in the Monte L. Bean Life Science Museum under accession numbers: *S.melanops* (BYU:1003048), *S.ciliatus* (BYU:1003050, BYU:267108) and *S.variabilis* (BYU:1003082, BYU:267107), (Table 1). Morphological vouchers were retained for *S.ciliatus* and *S.variabilis* collected in 2018 (BYU:1003050 and BYU:1003082, respectively). Total DNA was extracted from 40 mg liver samples using a DNeasy Blood & Tissue kit (Qiagen, Hilden, Germany). DNA concentration was measured by a Qubit Fluorometer (Thermo Fisher Scientific, Waltham, MA, USA). Libraries of each sample were created according to the Illumina Library prep protocol (Illumina 1003806 Rev. A) and sequenced on the Illumina HiSeq 2500 (Illumina, San Diego, CA, USA; Paired End 150 bp) at Brigham Young University’s DNA Sequencing Center (Provo, Utah, USA). We used FastQC (Andrews 2010) to assess quality of raw reads. Mitogenomes were assembled with Geneious v. 2021.2 (Biomatters Ltd., Auckland, New Zealand) using *S.fasciatus* as a reference genome (KX897946) and annotated with MitoAnnotator (Iwasaki et al. 2013). Raw reads and assembled genomes were deposited in NCBI's Sequence Read Archive (raw data) and GenBank nucleotide (assembled mitogenomes) databases (Table 1). Mitogenome maps were generated using Circos (Krzywinski et al. 2009). We included 35 rockfish mitogenomes, representing 27 species, in our phylogenetic analysis. We used MAFFT v. 7.475 (Katoh et al. 2002) to generate multiple sequence alignments for each of the 13 protein-coding genes and concatenated the alignments. We generated a Maximum Likelihood phylogeny with W-IQ-Tree (Trifinopoulos et al. 2016).

## Results

**Figure 2.**
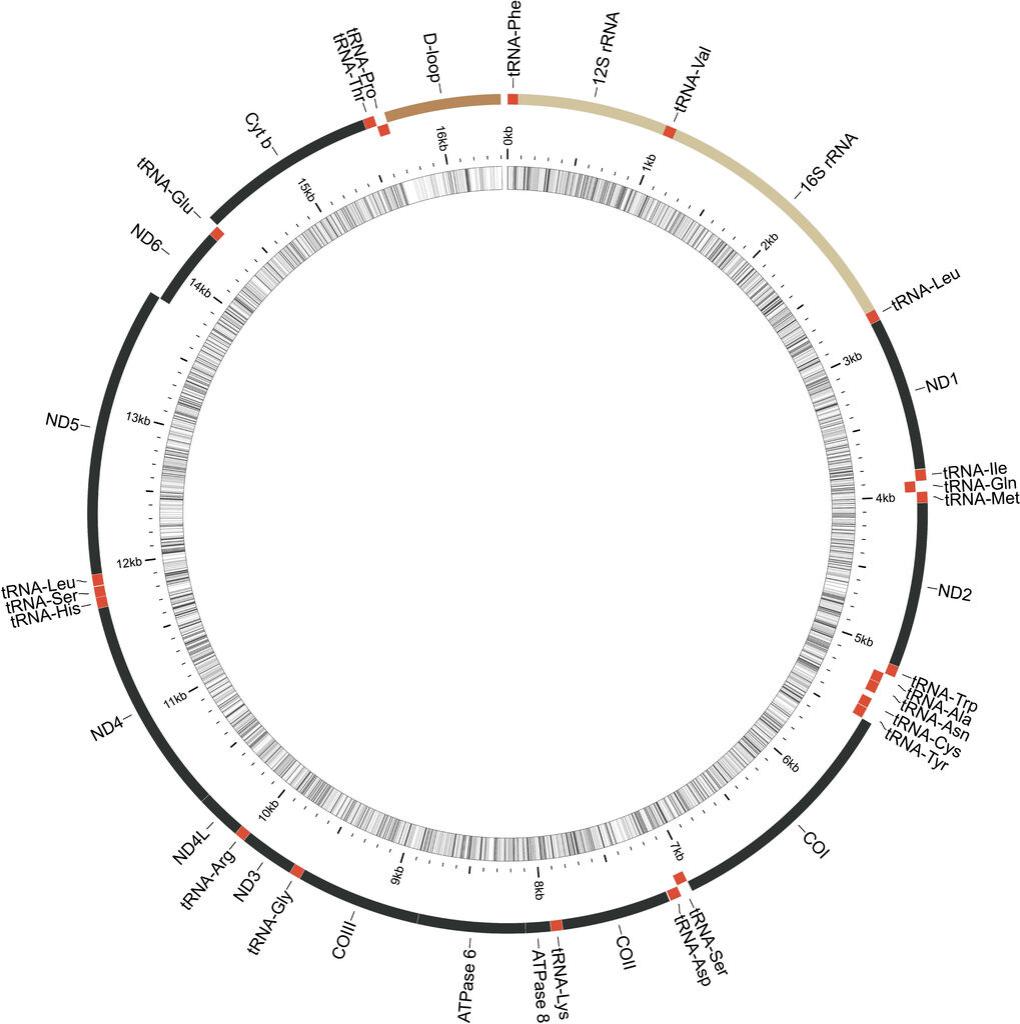
Mitogenome map of *Sebastesmelanops*. Outer circle illustrates order of genes, tRNAs, rRNAs and control region. Inner circle represents GC content with darker shades indicating higher GC content. *Sebastes melanop's* mitogenome consists of 13 protein-coding genes, 22 tRNAs, two rRNAs and one control region. Order is identical in *S.ciliatus* and *S variabilis* (mitogenome maps not displayed).

**Figure 3.**
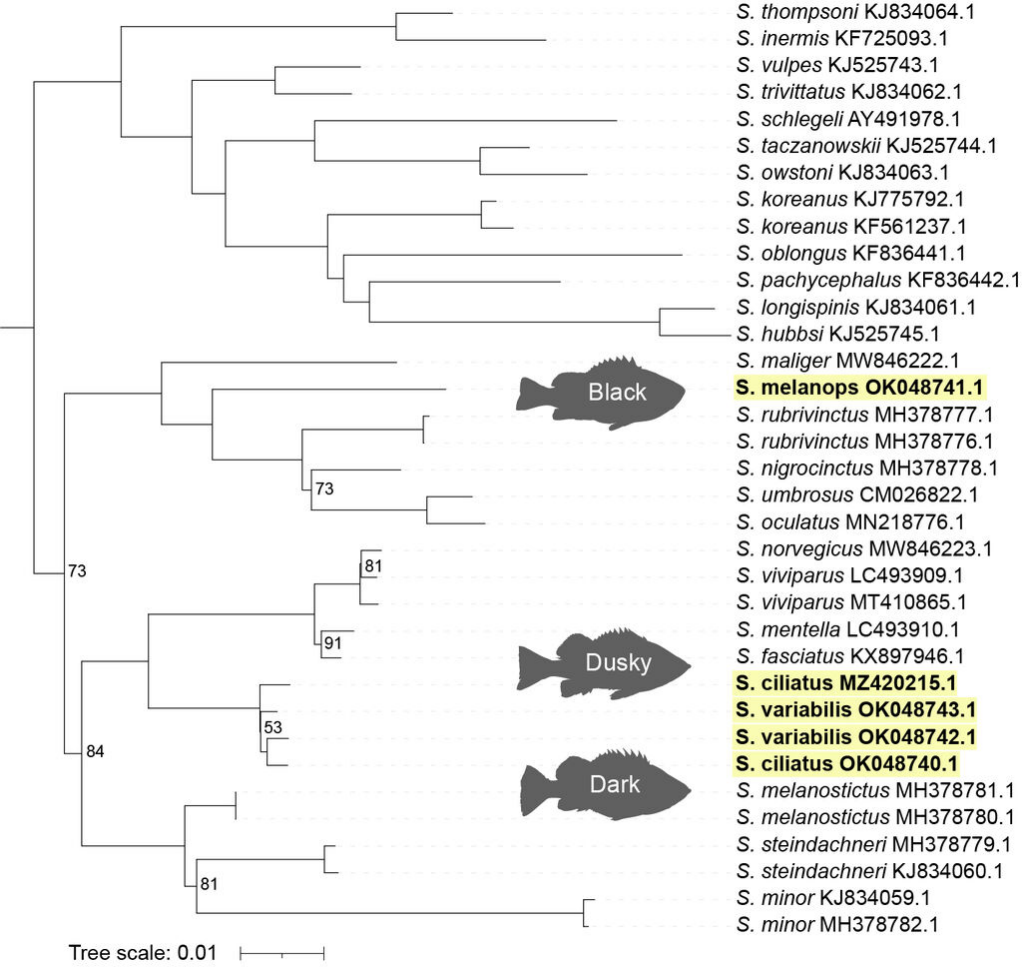
Phylogenetic tree inferred by Maximum Likelihood using W-IQ-Tree. Thirty-five *Sebastes* mitogenomes, representing 27 species, were used in the phylogeny. Ultrafast bootstrap values > 95 are not displayed and *Sebastiscustertius* (MT117231) was used as an outgroup, but is not shown.

The complete mitochondrial genome of *Sebastesmelanops* (OK048741) was 16,405 bp in length, *S.ciliatus* (MZ420215, OK048740) were both 16,400 bp in length and *S.variabilis* (OK048743, OK048742) were 16,400 and 16,401 bp, respectively, in length. Consistent with previous studies (Zhang et al. 2012, Sandel et al. 2018, Campbell et al. 2022), the control region’s length was highly variable because of repetitive DNA sequences (Fig. 2). The complete mitogenomes of *S.ciliatus* and *S.variabilis* were ~ 0.5% divergent. In comparison, the complete mitogenome of *S.melanops* was between 6.2% and 10.6% divergent with other members in its clade. In our phylogeny, *S.melanops* is sister to a clade including *S.rubrivinctus*, *S.nigrocinctus*, *S.umbrosus* and *S.oculatus*, whereas *S.ciliatus* and *S.variabilis* are sister to a clade including *S.norvegicus*, *S.viviparus*, *S.mentella* and *S.fasciatus*. We were unable to resolve the phylogenetic relationship between *S.ciliatus* and *S.variabilis* (Fig. 3).

## Discussion

Previous molecular analyses of allozymes, microsatellites and mitochondrial DNA have produced inconsistent results about the relationship of *Sebastesciliatus* and *S.variabilis* (*Tsuyuki et al. 1965, Seeb 1986, Orr and Blackburn 2004*). However, in these studies, it is unclear if these differences result from species-level or population-level differences. In addition, for the studies that used mitochondrial DNA, only a small portion of mitochondrial DNA was examined (Orr and Blackburn 2004). By using samples of *S.ciliatus* and *S.variabilis* from sympatric populations, as well as generating whole mitochondrial genomes, we provide new insight into the status of these species. Consistent with previous studies using partial mitochondrial sequences, we found minimal sequence divergence between *S.ciliatus* and *S.variabilis*. Assuming *S.ciliatus* and *S.variabilis* are distinct sister species, we would expect greater sequence divergence, as well as a monophyletic relationship in our phylogeny, with higher bootstrap values. Further research is needed. This research should include specimens from a wider range of locations across their geographical ranges, with both allopatric and sympatric populations, a suite of genetic markers (nuclear and mitochondrial), as well as ecological and morphological characteristics. Such information will be essential in resolving the complicated relationships between these two putative species.

## Figures and Tables

**Table 1.** Voucher, BioProject, BioSample, GenBank and SRA accession numbers for each sample of *Sebastes* used in the study.

Species	Voucher	BioProject	BioSample	GenBank	SRA
*S.ciliatus*	BYU:267108	PRJNA741690	SAMN20892472	OK048740	SRX11870776
*S.ciliatus*	BYU:1003050	PRJNA741690	SAMN20892468	MZ420215	SRX11870778
*S.melanops*	BYU:1003048	PRJNA741690	SAMN20892467	OK048741	SRX11870777
*S.variabilis*	BYU:267107	PRJNA741690	SAMN20892471	OK048742	SRX11870775
*S.variabilis*	BYU:1003082	PRJNA741690	SAMN20892469	OK048743	SRX11870779
